# Learning and retrieving holistic and componential visual-verbal associations in reading and object naming

**DOI:** 10.1016/j.neuropsychologia.2016.09.025

**Published:** 2017-04

**Authors:** Connor Quinn, J.S.H. Taylor, Matthew H. Davis

**Affiliations:** a MRC Cognition and Brain Sciences Unit, Cambridge, UK; b Department of Theoretical and Applied Linguistics, University of Cambridge, UK; c Department of Psychology, Royal Holloway University of London, Egham, Surrey, UK

**Keywords:** FMRI, Reading, Learning, Orthography, Consolidation

## Abstract

Understanding the neural processes that underlie learning to read can provide a scientific foundation for literacy education but studying these processes in real-world contexts remains challenging. We present behavioural data from adult participants learning to read artificial words and name artificial objects over two days. Learning profiles and generalisation confirmed that componential learning of visual-verbal associations distinguishes reading from object naming. Functional MRI data collected on the second day allowed us to identify the neural systems that support componential reading as distinct from systems supporting holistic visual-verbal associations in object naming. Results showed increased activation in posterior ventral occipitotemporal (vOT), parietal, and frontal cortices when reading an artificial orthography compared to naming artificial objects, and the reverse profile in anterior vOT regions. However, activation differences between trained and untrained words were absent, suggesting a lack of cortical representations for whole words. Despite this, hippocampal responses provided some evidence for overnight consolidation of both words and objects learned on day 1. The comparison between neural activity for artificial words and objects showed extensive overlap with systems differentially engaged for real object naming and English word/pseudoword reading in the same participants. These findings therefore provide evidence that artificial learning paradigms offer an alternative method for studying the neural systems supporting language and literacy. Implications for literacy acquisition are discussed.

## Highlights

• Generalisation of novel orthography shows componential processing in reading.
• Real and artificial words and objects rely upon the same neural systems.
• Different neural systems support reading novel orthography and naming novel objects.
• No evidence of whole-word cortical representations for artificial written words.
• Reduced hippocampal responses suggest overnight consolidation of artificial items.

## 1 Background

In recent years the education literature has settled upon phonics as the only evidence-based method of teaching reading (Torgerson et al., 2006, Wyse and Goswami, 2008). Indeed, in the UK, the Rose Review (Rose, 2006) recommended that synthetic phonics, which involves explicit instruction in letter-sound decoding and blending, should underlie early reading instruction. This provides children with the primary skill of being able to translate *print to sound*. Whole-word methods of reading instruction instead argue for the primacy of meaning in reading, with knowledge of letter-to-sound mappings being acquired through exposure to meaningful text. In this case the primary skill of reading should not be translating *print to sound*, but instead *print to meaning*. Correspondingly the focus of early learning in whole-word reading schemes is to recognise whole ‘sight words’, rather than decoding the letter-sound correspondences within each word. Thus, many are sceptical of whether, in line with the Rose Review, phonics should be taught “first and fast”. While experimental data has an important role to play in this activity, Wyse and Goswami (2008) note that very few naturalistic studies comparing different methods of reading instruction meet rigorous experimental standards. In this paper we consider whether laboratory studies of holistic and componential visual-to-verbal learning may offer a way to address educational questions in a controlled manner.

The distinction between recognising whole-words and decoding letter-by-letter in the educational literature is mirrored to a large extent by findings from cognitive research on reading. Cognitive models of reading, such as the Dual Route Cascaded (DRC) and triangle model, reflect the distinction between holistic and componential processing by suggesting that the meaning of a written word can be accessed in more than one manner (Coltheart et al., 2001, Plaut et al., 1996). For example, in the DRC model, words can be read componentially by decoding letter-by-letter (sub-lexical route), or can be mapped onto their pronunciations and meanings directly by recognising the whole word form (lexical route). It is the componential relationship between visual and phonological forms in alphabetic languages that means we can read pseudowords, e.g. ‘spape’, using our knowledge of letter-sound mappings. In contrast, to read an irregular word (e.g. ‘pint’) we must have whole-word knowledge to know that it does not sound similar to words that share the same orthographic components (‘mint’, ‘hint’, etc.). In the triangle model (Plaut et al., 1996) the mappings between written (orthographic) and spoken (phonological) forms are componential; this model does not contain whole-word, or lexical, representations of this information. However, in this model, the relationship between a familiar word's written form and its meaning is more holistic and item-specific, since the form-to-meaning mapping cannot be broken down into sub-components, at least for monomorphemic words (i.e. most monosyllabic words). Furthermore, this item-specific knowledge is proposed to be important for irregular word reading, helping them to be pronounced differently from similarly spelled regular words. Thus, both the DRC and the triangle model propose that reading involves both componential and whole-word knowledge, with the former being more important for pseudowords or less familiar words, and the latter more important for words, in particular those with irregular spellings.

Although both componential (sub-lexical) and holistic (lexical) processes are involved in skilled reading it is not clear how the relative importance of these skills changes as we learn to read. The goal of the present study was to advance our understanding of the initial stages of reading acquisition by exploring the neural basis of componential and holistic processing. To do so we compared learning to read an artificial alphabetic orthography with systematic symbol-to-sound mappings with learning names for novel objects with arbitrarily associated names.

### 1.1 Neural bases of holistic and componential processes in reading

The ventral occipito-temporal (vOT) cortex, including posterior and anterior fusiform, inferior temporal, and lateral occipital regions, has been suggested to play an important role in visual processing of orthographic information (Cohen et al., 2000, Cohen et al., 2002, Dehaene et al., 2002). A variety of evidence suggests that these visual processes are hierarchically organised with componential representations of individual letters and letter sequences in posterior temporal and occipital regions, and more holistic representations of whole words in anterior temporal regions (Dehaene et al., 2005, Taylor et al., 2013). For example, Mechelli et al. (2005) found that posterior fusiform activation was greater for pseudowords than for irregular words such as ‘pint’, whereas anterior fusiform showed the reverse profile. Likewise, Vinckier et al. (2007) showed a hierarchy of neural representations of letter strings in vOT: more posterior vOT activated for all stimuli (including consonant strings and false fonts), whereas mid- to anterior-fusiform regions were only activated for letter sequences that contained familiar letter combinations. In addition, Seghier et al. (2008) found that adult readers who were slower at reading pseudowords than irregular words showed additional activation in both left inferior parietal and left posterior occipito-temporal cortices, reflecting increased effort in componential reading processes. In contrast slower reading of irregular words was associated with increased activation in left anterior occipito-temporal and left ventral inferior frontal regions. These findings support the idea that posterior fusiform and occipito-temporal cortex process parts of words whereas anterior fusiform processes whole-word forms. Debate continues concerning whether this vOT hierarchy includes brain regions that uniquely contribute to reading (Dehaene and Cohen, 2011), or are shared with other domains in which visual and phonological information is associated, e.g. object naming (Price and Devlin, 2011).

In addition to these posterior occipito-temporal regions, a number of other brain areas have been shown to contribute to componential reading processes, as highlighted by contrasting pseudoword and word reading (see review and meta-analyses by Taylor et al. (2013), Cattinelli et al. (2013)). Pseudoword relative to word reading activates left inferior frontal and precentral gyri, which are involved in phonological output processes, left inferior parietal cortex, which may be involved in mapping letters to sounds, and left posterior occipito-temporal cortex, which may contribute to sub-lexical analyses of written word forms. The reverse contrast of word relative to pseudoword reading, capturing holistic reading processes, activates left middle temporal and angular gyri, regions which may support semantic processing (see Taylor et al. (2013) for discussion).

In summary the componential and holistic processes that underlie reading appear to be supported by different neural systems (holistic reading in anterior vOT regions, componential reading in posterior vOT, inferior parietal cortex, and inferior frontal gyrus). However, as discussed at the outset, the relative role of holistic and componential processes in *learning to read* is not clear. Experimental evidence of the relative contribution of these neural systems in the initial stages of reading instruction might therefore contribute to a scientific understanding of debates between phonic and whole-word approaches to reading acquisition.

### 1.2 Neural contributions to learning to read

There are two broad methods by which neuroscientists have studied the brain changes associated with the emergence of literacy (see Dehaene et al. (2015) for a review). The first of these is to explore neural activity in children at different stages of learning to read. Activation in vOT to words has been shown in young children in tasks involving sub-lexical processing such as single letter naming (Turkeltaub et al., 2008) and associating letters with sounds (Brem et al., 2010), but also for lexical tasks such as single word reading (Church et al., 2008). Furthermore, a meta-analysis of 40 imaging studies showed that both child and adult readers showed activation in left vOT, inferior frontal, and posterior parietal regions (Martin et al., 2015). However, there were also age-related differences: activation was more consistently observed in posterior fusiform regions for adult than child readers, possibly reflecting increased sensitivity in adults to the differences between letters and control stimuli. Tracking neural changes in a single group of children over four years, Ben-Shachar et al. (2011) showed that the sensitivity of left vOT to written words increased as reading improved, and that this was correlated with sight word naming accuracy but not with measures of pseudoword reading. Furthermore, the spatial extent of the cortical region sensitive to visual words increased as children got older before decreasing until reaching adult level. This changing response may reflect the region initially becoming more engaged for orthographic inputs before later becoming more efficient as specialisation takes place, following an inverted-u shaped profile (Ben-Shachar et al., 2011, Price and Devlin, 2011). Taken together, these results suggest that vOT regions become more sensitive to orthographic information with increased age/proficiency but it is not clear whether this change is linked to holistic or componential reading processes.

Parietal activation in children has primarily been shown in tasks involving mappings between visual words and sounds, (e.g., Bitan et al., 2006, Bitan et al., 2007a, Bitan et al., 2007b; Cao et al., 2006; Hoeft et al., 2007). For example children making spelling (orthographic) or rhyme (phonological) judgements about visually presented words showed increased activation in bilateral inferior/superior parietal lobules for spelling compared to rhyme judgements (Bitan et al., 2007a). Likewise Hoeft et al. (2007) found that activation in left inferior parietal lobes correlated with composite behavioural measures of phonics ability in children. Further evidence that parietal regions support the componential aspects of reading early in development comes from Cao et al. (2015) who compared adult and child English and Chinese speakers in a visual word rhyming task. Reading skill in English speaking children was correlated with activation in left inferior parietal lobule. The same was not true for Chinese speaking children, lending support to the idea that early reading in English, with its reliance on componential letter-sound mappings, engages left parietal regions more than logographic reading in Chinese readers.

One problem with studies comparing children and adults is that it can be difficult to distinguish neural changes due to increased proficiency from changes due to maturation. The second approach to studying literacy-related changes in the brain circumvents this problem by examining functional and structural changes in adults who learned to read later in life. Dehaene et al. (2010) found that, compared to illiterate adults, both adults reading from childhood and late-learners showed greater activation to written words in left fusiform gyrus as well as language regions such as left superior temporal sulcus and left inferior frontal gyrus. Furthermore, adults who learned to read later in life showed increased grey matter in angular gyri, dorsal occipital, middle temporal, supramarginal, and superior temporal gyri, in comparison to illiterate adults (Carreiras et al., 2009).

In summary, evidence from studies of beginning readers and ex-illiterate adults has shown increased contributions of vOT regions with increased reading skill. It remains unclear, however, whether these contribute to holistic or componential processing of written words. Evidence for componential processes seems to point to inferior parietal regions which might play a preferential role in initial stages of acquisition. This might be taken as consistent with the componential, phonics-based educational literature introduced at the outset which similarly suggests that initial stages of teaching should focus on componential decoding skills. One possible challenge however, is that these studies with children and adults have only explored relatively late stages of reading acquisition. It would be extremely difficult to attempt to scan children in their first months of literacy learning (in the UK, this would require scanning 4-year old children since reading instruction begins at that age). Therefore, in the present work we explore the initial stages of reading instruction for adults learning to read in an artificial orthography. To the extent that changes in vOT and parietal brain activity for holistic and componential learning parallel activation seen during reading development we may be confident in attributing neural changes to the balance of these two underlying processes.

### 1.3 Using artificial orthographies to study the early stages of literacy acquisition

Laboratory-based learning paradigms offer a valuable method to address questions about reading, allowing a degree of experimental control impossible to achieve in naturalistic learning situations. Here we review previous studies that provide methods for distinguishing holistic and componential learning of artificial stimuli. One such artificial language learning study taught two groups of participants to read a single set of stimuli with either alphabetic (componential) or logographic (holistic) mappings to phonology over the course of eight days (Mei et al., 2013). Imaging results showed increased left-lateralisation for the more componential orthography, particularly in posterior fusiform regions. Likewise, ERP studies have reported left lateralised fusiform responses for componential as opposed to holistic learning of artificial orthographies (Yoncheva et al., 2010, Yoncheva et al., 2015).

The current study adapts and extends the methods used by a previous study that compared the neural bases of learning holistic and componential visual-verbal mappings using artificial objects or artificial written words. Taylor et al. (2014a) taught participants to name artificial objects and to read words written in an artificial orthography, whilst neural activity was measured with fMRI. Imaging results showed that learning to name objects preferentially activated bilateral anterior fusiform gyri, whereas learning to read words activated bilateral inferior parietal cortices. Taylor et al. therefore suggested that anterior fusiform is associated with whole item visual-verbal associations whereas inferior parietal cortex is involved in componential visual-verbal mappings. However, this componential interpretation of parietal contributions remains controversial; Takashima et al. (2014) have suggested that extensive training on a small set of words written in Greek script provided fMRI evidence for holistic representations in brain regions close to the inferior parietal cortex (such as the angular gyrus, precuneus, and middle temporal gyrus). A key question from these results, then, is how, where and when do holistic representations of written words emerge?

### 1.4 The current study rationale

Here we set out to track the changes in neural activity that occur over the first two days of learning to read and name artificial words and objects. In doing so we build on previous work on the neural systems previously identified in *learning* holistic and componential visual-verbal associations. We extend this earlier work by asking: (1) do we see a similar neural dissociation during *retrieval* of holistic and componential visual-verbal associations. Furthermore, we ask whether neural activity associated with retrieval of written words offers evidence of the emergence of whole-word representations, (2) in the context of overnight consolidation and (3) by comparison with untrained words. Finally, we assess: (4) the extent to which neural activity associated with reading and naming artificial words and objects overlaps with regions involved in holistic and componential processing of real words, objects, and pseudowords. As we will explain below, these four elements substantially extend the findings of Taylor et al. (2014a) while using similar methods.

In the current study, we trained participants outside of the scanner to read artificial words written in an artificial orthography, and to name artificial objects. Critically, the artificial written words had a componential and systematic mapping between the visual (letters) and verbal (sounds) forms, whereas artificial objects had a holistic and arbitrary relationship between the visual and verbal forms. That is to say, if participants successfully learn the componential letter-sound mappings of the written words then they should be able to generalise this knowledge in order to read unfamiliar words. By contrast because the relationship between the visual and verbal forms of an object is arbitrary and holistic; it is not possible to name an unfamiliar object.

Whilst Taylor et al. (2014a) focussed on measuring neural activity during the learning of holistic and componential visual-verbal mappings by training participants during scanning, here we focus on activation during retrieval of knowledge previously acquired outside of the scanner. This design allowed us to dedicate more time to testing neural activity associated with reading or naming of trained items during scanning. We were also able to adopt an event-related fMRI design in which written words and objects were intermixed, in contrast to the blocked design used by Taylor et al. (2014a). This may be more sensitive to detecting activation differences between words and objects (Josephs and Henson, 1999) and increases the likelihood that any activation differences between reading written words and naming objects are due to the immediate demands of processing of componential and holistic visual-verbal associations, and not due to longer-term differences in the strategy adopted in different testing blocks.

In order to investigate the time-course over which holistic representations of written words may emerge we adapt the train twice, test once design used by Davis et al. (2009) to explore the neural effect of overnight consolidation for spoken words. Following the method used by Davis et al., half of the words and objects in the current study were learned on day 1 and the other half on day 2, with scanning taking place following day 2 training. This design allows an opportunity for overnight consolidation of day 1 but not day 2 items. In line with complementary learning systems accounts (Davis and Gaskell, 2009) we might anticipate differences between neural responses for items scanned following a night of sleep as compared to items learned on the same day as scanning. Using this design does not allow us to distinguish whether any consolidation effects come about due to the processes of sleep or due to time elapsed since learning. Nevertheless the design allows us to compare initial and longer-term changes during the earliest stages of learning to read. It will be for future research to determine the underlying cause(s) of any changes observed.

**Fig. 1:**
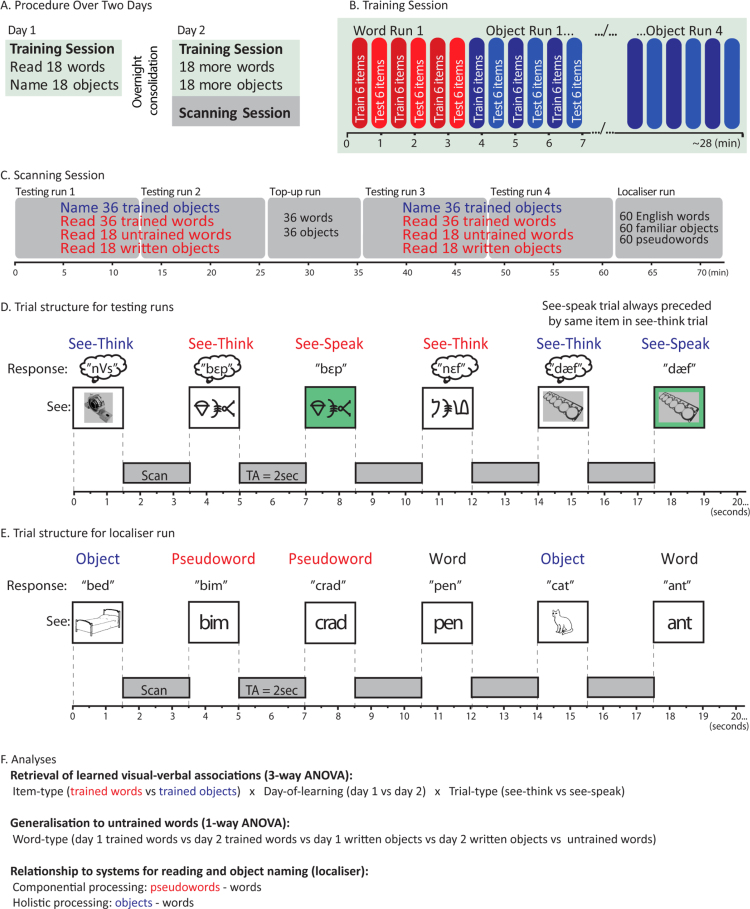
(A) Timing of training and testing procedures on the two days of the study. (B) Train/test structure used during the training sessions with alternating periods spent learning and being tested on words and objects. Within each run participants were trained then tested on 6 items at a time until all 18 items had been tested. (C) Timeline of fMRI scanning runs including testing runs, top-up, and localiser runs. In testing runs 1 and 2 participants were presented with each of the trained items twice in a see-think trial and once in a see-speak trial. Half of the untrained items were similarly presented across these two testing runs. Testing runs 4 and 5 followed the same structure with all trained items presented again, and the other half of the untrained items. (D) Time line showing the structure of the two trial types presented during the scanning runs: see-think and see-speak trials. Word and object trials were presented in a random order for each participant with the constraint that see-speak trials always followed a see-think trial for the same item. However, see-think trials were also presented in isolation so that activity for these two trial types can be separated at the analysis stage. See-speak trials were identical to the see-think trials except that the green background cued participants to say the appropriate word/object name aloud rather than covertly. (E) Timeline showing trial structure of the localiser run. Familiar words, objects, and pseudowords were presented onscreen. Participants read/named all items aloud during the silent interval between scans. (F) The goal of each of the reported analyses of the neuroimaging data and the conditions compared.

In addition to reading or naming all the trained words and objects from days 1 and 2, participants also read three sets of untrained artificial words during scanning. These conditions therefore permitted a comparison of trained and untrained words – allowing us to assess activation differences that might parallel those seen between English words and pseudowords. Two sets of these untrained words were the written forms of the object names learned on days 1 and 2. The spoken forms of these items were therefore familiar (trained) while the orthographic forms remained unfamiliar (untrained) (Fig. 1). This manipulation allowed us to determine if familiarity with the phonological form of a word (in the context of object naming) results in differential activation as compared to the third set of untrained words which were completely unfamiliar. For these comparisons, we can therefore assess the possibility (raised by research on spoken word learning, Davis and Gaskell, 2009) that holistic lexical representations are enhanced during overnight consolidation and hence that these effects may differ for words learned on day 1 or day 2. These comparisons would be very difficult to achieve in a naturalistic setting.

In order to validate the use of this laboratory-learning approach in relation to the brain systems ordinarily engaged for word reading and object naming we also included a functional localiser scan in which participants read English words and pseudowords and named familiar objects. This allowed us to ask whether the neural systems that support reading of artificial words and naming of artificial objects are the same as those used for real word reading and object naming. This may help us in ascertaining whether a neural distinction between holistic and componential processing applies equally in artificial and real reading and object naming.

In summary then we address four major questions in this study: 1) Are the same neural systems involved in the retrieval of holistic and componential visual-verbal associations as previously shown to support learning? 2) How does overnight consolidation impact on neural representations of recently learned words and objects? 3) What do comparisons of reading trained and untrained words suggest about neural systems for whole word representation and generalisation to pseudowords? 4) To what extent do the neural systems involved in reading and naming artificial words and objects overlap with the systems involved for real words and objects?

## 2 Materials and methods

### 2.1 Participants

25 right-handed native English speaking adults aged 18–40 took part in a study approved by the Cambridge Psychology Research Ethics Committee. No participants reported having dyslexia, speech, or language impairment or any pre-existing neurological condition that would preclude participation in functional MRI. Five participants were excluded due to poor performance during scanning (<20% correct on any condition) leaving 20 participants in the main analyses. An additional participant did not complete the functional localiser run and so the localiser analysis reports the results from 19 participants.

### 2.2 Experimental Stimuli

Three sets of 36 monosyllabic consonant-vowel-consonant (CVC) pseudowords were used in the experiment (e.g., “pag”, “zon”). Each pseudoword set was assigned to one of three conditions in a counterbalanced manner over participants: objects, words, and an additional set of untrained words (Appendix A). These pseudowords were constructed from the same set of 12 consonants (b, d, f, g, k, m, n, p, s, t, v, z) and four vowels (ɒ, ɛ, æ, ʌ) as used in Taylor et al. (2014a). Segment position was matched across stimulus groups with each consonant appearing three times each in onset and coda position while each vowel appeared 9 times in each item set. Each of the three stimuli sets was further split into two groups of 18 items to be trained on days 1 and 2. Unfamiliar visual symbols (artificial letters) were mapped to the 16 phonemes in a one-to-one manner meaning that the written forms of items had consistent letter-sound mappings (see Fig. 1D for examples). Spoken forms of the pseudowords were recorded by a female native English speaker in a soundproof booth and digitised at a sampling rate of 44.1 kHz.

Stimuli for the functional localiser were 120 monosyllabic items chosen from the updated Snodgrass and Vanderwart item set (Magnié et al., 2003). These were randomly assigned on participant-by-participant basis to appear either as objects for naming (60 pictures) or as words for reading (60 written words). This prevented potential priming effects that would occur if all items appeared as both words and objects for each participant. 98 of the 120 items (81.6%) had grapheme-to-phoneme correspondences that are classified as regular according to the DRC model of reading (Rastle and Coltheart, 1999, Appendix C), and the mean log frequency of the items based on the Zipf scale (Van Heuven et al., 2014) was 4.47 (*SD*=0.56), i.e. relatively high frequency. 120 monosyllabic pseudowords were generated from the ARC nonword database (Rastle et al., 2002) and were pairwise matched to these Snodgrass and Vanderwart items for letter length and orthographic neighbourhood size (S&V item: mean length (*SD*)=4.09 (0.82), orthN mean (*SD*)=8.30 (5.14); Pseudowords: mean length (*SD*)=4.20 (0.75), orthN mean (*SD*)=8.27 (4.39)). Snodgrass and Vanderwart items and pseudowords were further matched pairwise for initial phoneme as this factor has been reported to have the most impact on reading and naming latencies (Rastle et al., 2005). 60 of these pseudowords were randomly selected for each participant. Localiser items (words, pseudowords, and objects) were therefore matched at a group level but not for each individual participant.

### 2.3 Experimental procedure

The experiment used a train twice, scan once design in which behavioural and neural responses to items learned on day 1 and day 2 (hereafter day 1 and day 2 items) can be compared in a single scanning session performed on day 2 (Fig. 1A). Participants learned different items over two days and then completed a combined fMRI and behavioural testing session following training on the second day. As a consequence of testing only once this efficient scanning design removes effects of practice on neural responses (e.g., for a longitudinal design) and avoids the neural variability that would be caused by testing on two occasions or scanning two different groups of participants (Davis and Gaskell, 2009).

#### 2.3.1 Training

Training took place over two days with 36 spoken pseudowords being associated with 18 artificial written forms and 18 artificial object pictures on each day. Participants completed eight runs of training on each day (four each of word and object training) consisting of alternating word and object runs (Fig. 1B). Within each run 18 items were presented across 6 blocks that alternated between training and testing. During trials in the training block participants passively viewed the visual form of each item onscreen for 3500 ms and then heard the spoken phonological form 500 ms after the visual onset. Six items therefore appeared in each training block. During the testing blocks, the same six items appeared in a different order (Fig. 1B). The visual forms were presented and participants read/named the item aloud during the 3500 ms in which the item was onscreen. Responses were recorded and scored offline and were deemed correct if the participant's response included all three phonemes in the target item in the correct order. At the end of the training on day 2, participants completed a short practice session of the task used in the scanner with real words and objects.

#### 2.3.2 MR data acquisition

Functional magnetic resonance imaging data were acquired using a 3 T Siemens Trio scanner (Siemens Medical Systems, Erlangen, Germany) with a 32 channel head coil. Responses were recorded with a dual-channel MRI microphone (FOMRI II, Optoacoustics). Audio stimuli were processed using the Sensimetric EQ Filtering 2.1 software for presentation over Sensimetric S14 headphones in the scanner. Visual stimuli were presented using a monitor mounted at the rear of the scanner bore, viewed via an angled mirror attached to the head coil.

We used a rapid sparse imaging event-related design with a repetition time (TR=3500 ms) longer than the acquisition time (TA=2000 ms), which allowed a gap of 1500 ms during which spoken responses could be recorded in the absence of scanner noise. This silent period between scans meant that participants could hear their own voice when speaking, and additionally reduced the impact of motion-induced artefacts on the acquired images (Peelle, 2014, Perrachione and Ghosh, 2013). Each of the four word/object test runs involved acquisition of 195 images (including 6 initial dummy scans to allow for T1 equilibrium). Image acquisition consisted of 32 transverse oblique axial slices, angled to avoid the eyes. Each slice was 3 mm thick and consisted of a 64×64 matrix of 3×3 mm voxels. There was a 0.75 mm gap between adjacent slices such that the total image volume allowed for whole brain coverage including the cerebellum, except for a few cases in which the very top of the parietal lobe was not covered. To assist in anatomical normalisation, we also acquired a T_1_-weighted structural volume using a magnetisation prepared rapid acquisition gradient-echo protocol (repetition time=2250 ms, echo time=2.99 ms, flip angle=9°, 1 mm slice thickness, 256×240×192 matrix, resolution – 1 mm isotropic).

#### 2.3.3 Scanning procedure

The scanning session consisted of 6 scanning runs lasting 72 min in total (Fig. 1C). Four of these runs tested artificial word reading and object naming. After two of these runs, participants completed a ‘top-up’ run where they were reminded of the items in the same manner as in the training sessions (i.e. paired presentations of visual and verbal forms of each item). This allowed another opportunity to learn the items and so increased the number of correct responses included in the analysis. However, neural data from this top-up run will not be reported in this manuscript. A functional localiser run that involved reading real words and pseudowords and naming real objects completed the scanning session. At the start of scanning a high-definition MPRAGE structural scan image was also acquired (see above for details).

Participants completed four reading/naming runs of 11 min duration while in the scanner (Fig. 1C). Each run contained 9 testing blocks of 63 s each with a rest period of 10.5 s between blocks. 18 trials appeared in each block of testing, made up of 12 see-think trials and 6 see-speak trials. All 36 trained words and 36 trained objects, along with half of the untrained words (n=18) and half of the written forms of trained objects (n=18) were split to appear across runs 1 and 2. The remaining half of the untrained words and written objects appeared in runs 3 and 4, along with a second presentation of all 36 trained words and 36 objects. Consequently untrained items were not repeated and so remained novel, while participants had two opportunities to name each of the trained items.

Critical to our design was that half of the see-think trials were followed by a see-speak trial in which the same item was presented (Fig. 1D). During see-think trials the items appeared on a white screen and participants were instructed to recall but not articulate the spoken form. For the see-speak trials items appeared on a green screen and participants were instructed to say the phonological form of the item aloud. Each trial lasted 3500 ms starting with a visual item presented for the first 1500 ms followed by a single functional brain volume being acquired in the remaining 2000 ms. Including see-think and see-speak trials was important to the design for several reasons. First, the time between scans was not long enough for participants to read a novel word and say it aloud. As the see-speak trials always followed immediately after a see-think trial participants had already retrieved the item pronunciation on the previous trial and could articulate its spoken form in the short period between scans. Second, this design ensured that the majority of trials were not affected by head movements due to articulation (as there were double the number of see-think trials as see-speak trials), and prevented anticipation of articulation on the subsequent trial, since participants could not predict whether a see-think trial would be followed by a see-speak trial. Third, as articulation only took place on see-speak trials, subtraction of see-speak from see-think trials will remove activation associated with articulation, and reveal activation associated with covert phonological retrieval on see-think trials. Finally, it is possible that activation differences between words and objects may in part be driven by visual differences between these two types of stimuli. As the same visual form was presented on successive see-think and see-speak trials, subtraction of see-speak from see-think trials may also reduce the impact of these visual differences. We return to this issue in the discussion.

During the localiser task participants were presented with 60 items in each of three conditions: written English words, pseudowords, and real objects. The same event-related sparse imaging design was used (TR=3500 ms, TA=2000 ms). Items were randomised and appeared onscreen for the 1.5 s of silence between scans using the same block structure as above; a 63 s block containing 18 trials followed by 10.5 s rest. 186 EPI images were acquired (~13 min scanning time).

#### 2.3.4 MRI preprocessing

Image processing and analysis of all EPI data were performed using SPM8 (Wellcome Trust Centre for Functional Neuroimaging, London, UK) in conjunction with AA software version 4 (Cusack et al., 2014). The first six volumes of each scanning run were discarded to allow for equilibration effects. Images for all scanning runs for each participant were realigned to the first image in the first scanning run (Friston et al., 1995) and the resulting mean image was co-registered to the T1 structural image. Normalisation of structural images to standard MNI space was calculated using tissue probability maps (Ashburner and Friston, 2005), and these warping parameters were then applied to all functional images for that participant. Normalized functional images were resampled to 2 mm isotropic voxels and spatial smoothing was applied using a kernel full-width-half-maximum of 8 mm. For the functional imaging analyses described below we used an event-related analysis implemented in the SPM8 software. Accordingly, event times were convolved with the SPM8 canonical hemodynamic response function following the recommendations of Perrachione and Ghosh (2013). Movement parameters estimated at the realignment stage of preprocessing were added as regressors of no interest. All analyses used a voxelwise threshold of p<0.001 combined with cluster extent-based FWE-corrected threshold of p<0.05 unless otherwise stated.

#### 2.3.5 Artificial words and objects analysis

First level models were constructed from all event types seen during testing runs (see-think and see-speak events for each of 7 conditions – words day 1, words day2, objects day 1, objects day 2, written objects day 1, written objects day 2, untrained words). The events were additionally split according to whether or not the response for each trial was correct or incorrect, leading to 28 event types in the first level model. Although all event types seen during testing were modelled, second level analyses focussed only on trials in which participants responded correctly, to ensure a fair comparison between conditions even if differences in accuracy were observed. In order to assign accuracy to see-think trials (in which there was no behavioural response) we assumed that accuracy in the see-speak trials could be applied to the corresponding see-think trials for that same item.

A second level model was constructed in SPM using 8 conditions derived for trained items that were responded to correctly involving three factors: trial-type (see-think vs see-speak), item type (words vs objects), and day of learning (day 1 vs day 2) (Henson and Penny, 2003, Technical Report). To assess neural responses for generalisation items (which were only presented in written form), a further second level model was constructed in which we compared responses for trained words (day 1 vs day 2), with the written form of object names (day 1 vs day 2), and untrained words leading to a 5-level factor that was crossed with trial-type (see-think vs see-speak). F-contrasts were constructed to identify significant main effects and interactions and, where significant effects were found, t-contrasts were used to explore the specific effects.

#### 2.3.6 Functional localiser analysis

We used an overt reading/naming task in order to overcome the challenge posed by Price and Devlin (2011) that passive viewing of words may induce greater covert naming than passive viewing of objects. To reveal the neural systems involved in holistic as opposed to componential visual–verbal processing of real world stimuli, we used the contrast [objects – words]. To ensure that this comparison revealed engagement of different neural representations, as opposed to differences in processing effort, this contrast was conducted after taking response time differences into account, using the approach proposed by Taylor et al. (2014b). This involves building a regression model that includes one parametric modulator to model the effect of RT on BOLD signal (irrespective of condition), and additional parametric modulator(s) representing the different stimulus conditions. Activation associated with this second parametric modulator then reflects the differences in neural response between conditions over and above effects due to response time differences.

The contrast [pseudowords – words] was included to reveal the neural systems involved in componential as opposed to holistic mapping between visual and verbal representations. Unlike the contrast between words and objects, we do not partial out the effects of response time when comparing words and pseudowords. Following the framework of Taylor et al., (2014b) both words and pseudowords should engage the same reading related brain regions, but pseudowords take longer to read aloud, and should therefore drive greater activity in these regions due to greater processing effort (c.f. Taylor et al., 2013, Taylor et al., 2014b). As this contrast was intended to reveal additional processing effort during pseudoword reading, response time differences between words and pseudowords were not taken into account when conducting this contrast.

Reading familiar words is an automatic process that is relatively effortless compared to both naming objects and reading pseudowords, leading to much shorter response times for the former than either of the two latter conditions. Given this, it is perhaps not surprising that contrasts of both [words – objects] and [words – pseudowords] showed activation throughout the default mode network (Gusnard and Raichle, 2001, Raichle, 2015). We therefore chose not to use these contrasts as these regions are unlikely to contribute directly to word reading (see Table 2 for details of these contrasts).

## 3 Results

### 3.1 Artificial words and objects during training

**Fig. 2:**
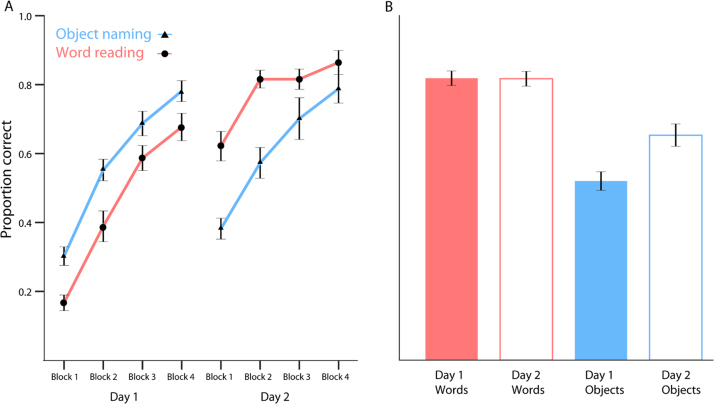
(A) Mean accuracy for Object Naming (blue) and Word Reading (red) during the four blocks of training on each day, error bars show +/−1 standard error of the mean after between subject variance removed suitable for repeated measures comparisons (cf. Loftus and Masson, 1994). (B) Accuracy in the scanner on day 2 while participants read/name items from both day 1 and day 2. Error bars as in panel 2 A.

Due to problems with audio recording equipment, responses for 4 participants were not collected during all of the training runs. For the remaining 16 participants we had full data from both days of training. These were scored for accuracy and entered into behavioural analysis of the training runs. Reading accuracy was better for words learned on day 2 than on day 1, whereas accuracy at naming objects was similar for day 1 and day 2 items (Fig. 2A, Table 1). Data were entered into a repeated measures ANOVA with the factors day of training (day 1 or day 2) and item type (word or object). There was no overall effect of item type on performance, *F*(1, 15)=0.5, ns. The effect of day was significant with day 2 items showing higher performance than day 1 items, *F*(1, 15)=8.86, ŋ_p_^2^=0.371, *p*<0.01. Furthermore there was an interaction between item type and day, *F*(1, 15)=8.81, ŋ_p_^2^=0.370, *p*=0.01, with performance for objects remaining similar on both days but with improved word reading performance on the second day.

### 3.2 Results during scanning

#### 3.2.1 Real words and objects in the localiser

**Fig. 3:**
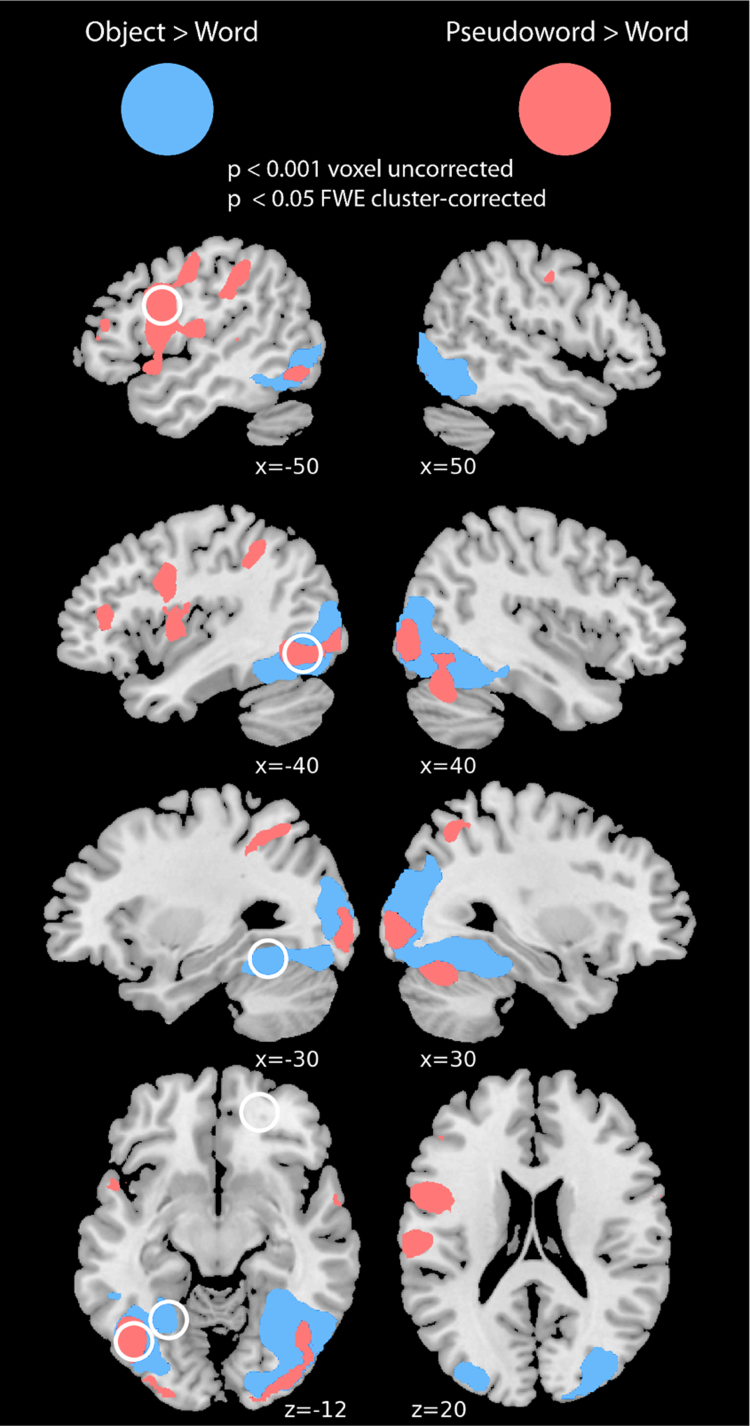
Brain regions showing differential activation for contrasts of interest from functional localiser displayed on the MNI standard brain. Red=[pseudowords – words], blue=[objects - words]. Slices show activations at p<0.001 voxel-wise uncorrected and p<0.05 FWE cluster corrected. White circles indicate approximate locations of regions of interest defined from the functional localiser.

Naming performance in all three conditions was very high (Table 1) but response times were faster for words than objects, t_p_(18)=21.11, p<0.001, and for words than pseudowords, t_p_(18)=8.32, p<0.001. As described in the methods, the contrast [objects – words] was conducted after taking between-condition differences in response time into account. This was to ensure that we could localise regions more engaged by holistic as opposed to componential processing rather than regions that responded more strongly to object naming as it was more effortful than word reading. Objects showed more activation than words in bilateral inferior temporal gyrus, as well as middle and inferior occipital gyri (Fig. 3, Table 2). We next contrasted pseudoword with word activation. As explained before, we did not account for response time differences in this analysis, since we wanted to observe neural activity associated with more effortful componential reading of pseudowords compared to reading of words (see methods and Taylor et al., 2014b). Pseudowords activated bilateral middle and inferior occipital gyri, left superior temporal gyrus, bilateral precentral gyri, bilateral postcentral gyri, left supplementary motor area, and left inferior frontal gyrus (triangularis), more than words (Fig. 3, Table 2).

In order to compare activation for real words and objects with the artificial words and objects we constructed four spherical regions of interest based on peaks found in the functional localiser that corresponded most closely to peaks found in a meta-analysis of word and pseudoword reading (Taylor et al., 2013). The ROIs had a 10 mm radius and were centred on: (1) left anterior fusiform gyrus (−28, −50, −16), determined by exploring sub-peaks of the left occipital/temporal cluster reported for objects – words in Table 2, (2) left posterior vOT (−42, −60, −8), (3) left superior parietal lobe (−26, −60, 54) and (4) left precentral gyrus (−54, 4, 26). ROIs (2), (3) and (4) were chosen from peak locations for activation during pseudoword – word reading in Table 2 (including a sub-peak of the cluster in superior temporal and frontal regions). These coordinates are respectively 17.2 mm, 7.48 mm, 6.63 mm, and 11.49 mm distant from comparable peaks in Taylor et al. (2013).

#### 3.2.2 Reading artificial words and naming artificial objects during scanning

Accuracy was higher for reading words than naming objects (Fig. 2B, Table 1). Whilst words from days one and two were read with equivalent accuracy, objects learned on day 1 were named less accurately than objects learned on day 2. A repeated measures ANOVA confirmed that accuracy was higher for reading words than naming objects, *F*(1, 19)=37.535, ŋ_p_^2^=0.664, *p*<0.001, and higher for items trained on day 2 compared to day 1 items, *F*(1, 19)=5.939, ŋ_p_^2^=0.238, *p*=0.025. There was also an interaction between day of training and item type, *F*(1, 19)=8.657, ŋ_p_^2^=0.313, *p*=0.008. Follow-up tests confirmed that naming accuracy for day 1 objects during scanning was significantly worse than for recently learned day 2 objects.

In the scanning session, in addition to reading the artificial words that were trained on days 1 and 2 (visually and phonologically familiar), participants also read the written forms of the object names trained on days 1 and 2 (visually unfamiliar, phonologically familiar), as well as a set of completely novel untrained words (visually and phonologically unfamiliar). These 5 conditions were entered into a one-way repeated measures ANOVA, allowing us to ask whether visual learning, phonological learning, and/or a period of offline consolidation leads to improved reading performance. Accuracy in all of these conditions was very similar (Table 1) with no significant difference between any of the conditions, *F*(4, 76)=0.694, ns.

#### 3.2.3 Whole-brain imaging analyses

**Fig. 4:**
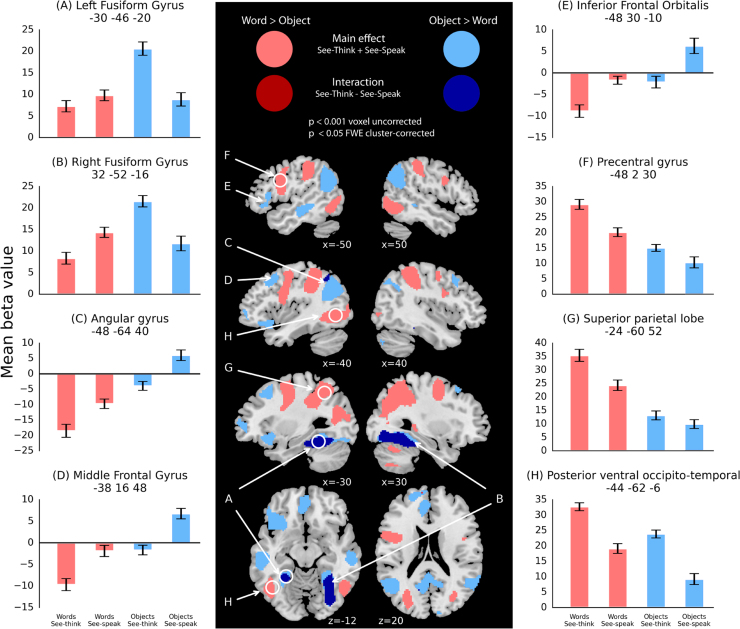
Brain regions showing differential activation for contrasts of interest for the artificial words and objects in see-think and see-speak conditions. Pale red=[words [see-think+see-speak]−objects [see-think+see-speak]], pale blue=[objects [see-think+see-speak]−words [see-think+see-speak]], dark red=[words [see-think − see-speak]−objects [see-think − see-speak]], dark blue=[objects [see-think − see-speak]−words [see-think − see-speak]]. NB: Dark red activation doesn’t reach statistical significance in this panel. Numbered plots show response profiles for contrasts versus the (unmodelled) resting baseline at peak locations labelled in the activation maps. White circles indicate approximate locations of regions of interest defined from the functional localiser (shown in Fig. 3).

To examine neural activity associated with reading trained words versus trained objects, and for items learned on days 1 and 2, we conducted a 2×2×2 repeated measures ANOVA to compare the effect of item type (word or object), day of learning (day 1 or day 2) and trial type (see-think or see-speak). We first examined the main effect of item type (words versus objects), collapsed across day of learning and trial type. The contrast words>objects showed extensive activation in bilateral parietal cortices, as well as peaks in middle and inferior occipital gyri (posterior vOT regions), right supramarginal, bilateral precentral, and bilateral middle frontal gyri, cerebellum, left supplementary motor area, and right hippocampus. (Fig. 4, pink, Table 3).

The reverse contrast objects>words revealed clusters in bilateral fusiform gyri (anterior vOT regions), bilateral angular gyri, left middle occipital cortex, left precuneus, left middle and anterior cingulate cortices, right inferior parietal cortex, bilateral superior and middle frontal gyri, left inferior frontal gyrus, left middle temporal gyrus, right cerebellum, right inferior temporal gyrus, right calcarine fissure, and left superior temporal pole (Fig. 4, light blue, Table 3).

As discussed in the methods, the main effect of word reading versus object naming does not rule out the possibility that low-level visual differences (such as differences in visual complexity, or retinal extent) between words and objects may be driving differences in activation. We therefore examined the interaction between item and trial type to reveal changes in object versus word retrieval-related activity during repetition suppression (i.e. additional activity for see-think trials, compared to the see-speak trials that immediately followed and that contained the same visual form). The contrast (words [see-think − see-speak]>objects [see-think − see-speak]) revealed no clusters at an FWE cluster-corrected threshold of p<0.05 (Fig. 4, Table 3). However, the contrast (objects [see-think − see-speak]>words [see-think − see-speak]) revealed clusters of activation in bilateral fusiform gyri and left cuneus (Fig. 4, dark blue, Table 3). As shown in Figs. 4A and 4B differential activity in left and right fusiform gyrus for objects compared to words is significantly greater during see-think than during see-speak trials. This might suggest that fusiform activity for objects is associated with initial identification and/or retrieving their names rather than being due to low-level visual differences. We will return to this point in the discussion.

The main effect of day showed no clusters surviving correction at whole-brain level. In addition, there was no interaction between day and item or trial type. The main effect of trial type was not of particular interest in this study, but is reported for similar, previous data in Taylor et al. (2014a).

#### 3.2.4 ROI analyses based on the real objects and words localiser

1) The ROIs defined in the localiser allow us to ask whether activation for trained artificial words and objects overlapped with that for real words and objects. Four regions of interest were defined based on the localiser, one in left anterior fusiform where objects show more activation than words, and three showing greater activation for pseudowords than words in left posterior vOT, left precentral gyrus, and left superior parietal lobe (the white circles in Fig. 3 show where the ROIs were defined while the white circles in Fig. 4 show how the ROIs relate to the whole-brain activation for the artificial words and objects.). In each ROI we conducted a 2×2 repeated measures ANOVA, with the factors item type and trial type, collapsed across day (Table 4). In the anterior fusiform ROI where real objects showed greater activation than real words, artificial objects also showed greater activation than artificial words and this was more pronounced for see-think than see-speak trials (similar to the interaction profile plotted in Fig. 4A). In the left posterior vOT ROI (similar to Fig. 4H), the left precentral ROI (similar to Fig. 4F) and in the left superior parietal ROI (similar to Fig. 4G) we saw activation consistent with contributions to reading artificial words with a main effect of item-type (words>objects), and of trial type (see-think>see-speak) but no interaction between these factors. Thus, ROIs which showed more activation for English pseudowords than words, also showed more activation for artificial words than objects, consistent with contributions to componential reading processes. Our confidence that these left hemisphere activation differences were driven by processes involved in recalling spoken from visual forms, as opposed to purely visual differences, is greater for objects in anterior fusiform, than for words in posterior vOT, precentral gyrus, and superior parietal cortex.
2) We used these same four ROIs to examine whether visual or phonological familiarity influenced neural activity during word reading. In each ROI, we conducted a one-way repeated measures ANOVA with the five word reading conditions (collapsed across trial-type): trained artificial words from day 1 and day 2, the written forms of artificial objects trained on days 1 and 2, and a set of untrained artificial words. This comparison allows us to ask whether visual and phonological familiarity (as for the trained words), or purely phonological familiarity (as for the written objects), or a period of offline consolidation (as for the day 1 items) is necessary to support more word-like representations. There was no significant difference between the five conditions in any of the four regions of interest (Table 4).

At the suggestion of a reviewer we additionally analyse these comparisons based on (1) whether the written words are visually familiar (trained words from days 1 and 2) or unfamiliar (written forms of objects from days 1 and 2, as well as untrained words), and (2) whether the written words are phonologically familiar (trained words from days 1 and 2 as well as written forms of objects from days 1 and 2) or unfamiliar (untrained words). Comparison of these conditions in each of the four regions of interest confirm no difference due to either visual or phonological familiarity (Anterior fusiform: visual familiarity, t(19)=1.314, ns. Phonological familiarity: t(19)=0.581, ns; posterior vOT: visual familiarity, t(19)=0.358, ns. Phonological familiarity: t(19)=1.406, ns; Superior parietal: visual familiarity, t(19)=0.775, ns. Phonological familiarity: t(19)=0.961, ns; Precentral gyrus: visual familiarity, t(19)=0.329, ns. Phonological familiarity: t(19)=1.441, ns).

#### 3.2.5 Hippocampal ROI analysis

**Fig. 5:**
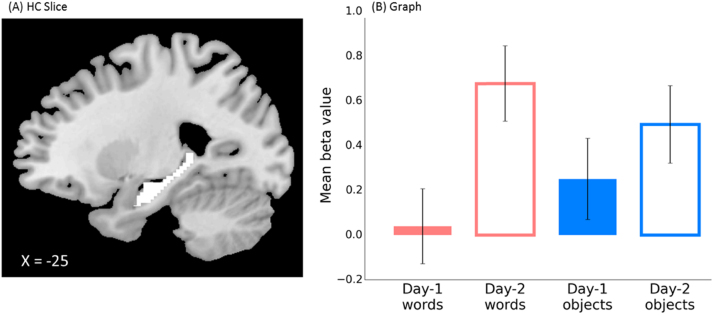
(A) Location of a bilateral hippocampus ROI defined using the AAL template brain. (B) This region showed differential activation (graphed as the contrast of activation versus an unmodelled resting baseline) for novel words and objects learned on days 1 and 2, collapsed across see-think and see-speak conditions and left/right hippocampus. Red= words, blue= objects, solid bars= day 1 items, empty bars= day 2 items.

Complementary learning systems (CLS) accounts of word learning (Davis and Gaskell, 2009) suggest a key role for the hippocampus, and indeed previous studies have shown changes in hippocampal responses to recently learned spoken words (Breitenstein et al., 2005; Davis et al., 2009; Takashima et al., 2014). As there is strong a priori reason to expect an effect of day of learning on hippocampal activity, regions of interest analyses were conducted using two separate AAL masks for the left and right hippocampi. Activation values were combined across see-think and see-speak trials and entered into a 3-way repeated measures ANOVA with factors day of training (day 1 or day 2), item type (word or object) and lateralisation (left or right hippocampus). Hippocampal activation was greater for day 2 than day 1 items, *F*(1, 19)=4.758, ŋ_p_^2^=0.20, *p*=0.042. There was no main effect of laterality, *F*(1, 19)=0.571, ns, or item type, *F*(1, 19)=0.049, ns, and no interactions reached significance (Fig. 5).

## 4 Discussion

Both educational and cognitive perspectives on reading have highlighted a critical distinction between holistic and componential processing; i.e., between recognising whole-word forms and decoding words letter-by-letter. Here we have compared an artificial learning paradigm to object naming and word reading of familiar, real language stimuli in order to identify the neural systems that support holistic and componential visual-verbal mappings and their acquisition. Our findings combine to demonstrate both behavioural and neural dissociations between holistic and componential mappings, which we will discuss in turn. We will start by summarising behavioural evidence for this distinction as shown by differences in learning profiles and generalisation, before moving onto discuss ventral occipito-temporal, parietal, and frontal contributions to reading and naming of both real and artificial items. We will conclude by returning to the educational issues that we introduced at the outset and consider the broader implications of our findings.

### 4.1 Behavioural results show holistic and componential learning

Behavioural results during training confirm that learning to read artificial words involved componential learning whereas learning to name artificial objects involved holistic learning. Participants become better at naming objects across four runs of training on day 1 but when they return on day 2 to learn 18 more objects their learning profile was essentially the same as on day 1. This pattern is due to the holistic and arbitrary relationship between the visual and verbal form of an object; knowledge of object-names from day 1 does not help to name objects learned on day 2. In contrast, the componential and systematic relationship between the visual and verbal forms of written words means that letter-sound mappings learned on day 1 are also effective in supporting reading of items learned on day 2. Hence, reading performance at the start of day 2 is substantially better than at the start of day 1.

The distinction between holistic and componential learning is further borne out by behavioural performance during scanning. Participants were significantly worse at naming objects learned on day 1 compared to those learned on day 2 – due either to forgetting of more distantly learned object names or interference from object names learned immediately prior to scanning on day 2. The present data does not distinguish these two possibilities (Anderson, 2003, Mensink and Raaijmakers, 1988). Whichever explanation we invoke, however, interference or forgetting arises from the holistic and arbitrary nature of visual-verbal mappings for object names; items learned on day 2 do not support, and might even interfere with, items acquired on day 1. In contrast, reading performance in the scanner was equally good for words learned on both days. Thus, the significant interaction between day and item type is again consistent with componential knowledge in reading words aloud; since items learned on day 2 contained the same letter-sound correspondences, this knowledge supported successful reading of words learned on day 1. Finally, the ability of participants to read untrained words accurately further demonstrates their ability to generalise this letter-sound knowledge to novel written words.

Hence our participants have acquired the ability to decode written words. This is the same skill as is taught to beginning readers through phonics instruction. These findings therefore support our use of functional neuroimaging of artificial language learning to explore the neural basis of learning to read. To the extent that neural activity overlaps for real and artificial items, we can further argue for parallels between the processes that support skilled reading/naming, and processes recruited for reading/naming our artificial materials. This will be the focus of the next two sections of discussion.

### 4.2 VOT contributions to reading and naming

A variety of evidence reviewed in the introduction suggests hierarchical organisation of visual processing in vOT regions. Results of the functional localiser to some extent support these proposals as the componential reading contrast (pseudowords>real words) showed activation in lateral and posterior vOT regions (replicating Mechelli et al. (2005); and others, see Taylor et al. (2013) for a meta-analysis). However, the holistic contrast of real objects>real words also showed activation throughout vOT, including posterior as well as mid- and anterior vOT regions. This finding appears more compatible with views of vOT specialisation that propose association with phonological representations as a key factor (Price and Devlin, 2011) rather than specialisation for alphabetic forms (Cohen et al., 2000, Dehaene and Cohen, 2007, Dehaene and Cohen, 2011). Our use of an overt naming task might be critical in explaining this observation. Covert reading/naming may induce greater phonological processing for word reading than object naming since phonological access is automatic for written words (Hagoort et al., 1999, MacLeod, 1991, Price et al., 1996, Song et al., 2010, Twomey et al., 2011). Nonetheless, we also observed that mid- and anterior vOT regions showed an additional response to objects than to words consistent with a contribution to processing holistic visual forms in hierarchically higher levels of the vOT.

Activation for the artificial words and objects matches the hierarchical organisation of vOT responses seen both in the localiser and in previous literature. We observed greater activation for reading artificial words than naming artificial objects in bilateral posterior vOT, whereas the reverse profile of greater activation for naming artificial objects than reading artificial words was observed in bilateral anterior vOT. Furthermore, this differential response in anterior vOT interacted with trial type, such that object>word activation was more pronounced for see-think than see-speak trials.

We preceded see-speak trials with a see-think trial for the same item primarily for pragmatic reasons: (1) it avoids articulation on the majority of trials, reducing head movement, because see-think trials occurred twice as often as see-speak trials (2) it would otherwise have been difficult for participants to produce item names in the short silent interval between two scans, (3) it permits separation and comparison of neural activity during covert (see-think) and overt (see-speak) articulation. That participants were able to respond fast enough on see-speak trials can be seen as a form of behavioural priming by which articulation of an item name is faster if it has been presented on an immediately preceding trial. Studies of neural repetition suppression have shown reduced activity for repeated items in a similar anterior fusiform region to that showing repetition suppression for artificial objects but not written words in the present study (Fig. 4A, cf. Kherif et al., 2011; Glezer et al., 2009). In previous work we have argued that the reduction in activity on see-speak compared to see-think trials reflects the fact that phonological retrieval primarily occurs on see-think trials (Taylor et al., 2014a). Hence, we proposed that anterior fusiform plays a greater role in processing holistic object-name associations than componential written word pronunciations. However, at the suggestion of reviewers, some more careful consideration leads us to acknowledge that there may also be visual contributions to repetition suppression. It is possible that anterior fusiform regions contribute to visual configural processing unique to objects and that this process, instead of, or as well as, phonological retrieval, is reduced on repeated trials (Vuilleumier et al., 2002, James et al., 2002). While other studies were able to rule this out (for example, Kherif et al. (2011) showed repetition suppression following pairs of non-identical pictures with the same name), our paired presentations involved the same visual form as well as the same name. Further investigations using names for objects that are depicted in multiple different pictures, and/or written words in multiple fonts, might help assess whether anterior fusiform is primarily involved in holistic relative to componential visual configural processing, or in the retrieval of holistic rather than componential visual-to-verbal associations.

One effect that we failed to see for the artificial orthography, which we had anticipated based on findings for reading real words and pseudowords was differential activation for trained versus untrained words. This null effect is notable considering the comparison involved 216 trials for trained words with 216 trials for untrained words (108 trials for the written forms of objects and 108 for completely untrained words), while the localiser showed differences between words and pseudowords with only 60 trials of each. This outcome, in conjunction with frontal and parietal activation for trained words relative to objects that we will discuss subsequently, might suggest that trained words were still being read componentially. It may be the case that our design included an insufficient number of training presentations (4 presentations of each word) to produce whole-word representations and that more intensive or longer-lasting training is required in order to generate holistic representations for written words in an artificial orthography. Future work will address this possibility. It might also be the case that adding irregular spelling-sound mappings would increase the necessity for these whole word representations. Note however, that cognitive models of reading, such as the DRC model, propose that whole-word representations develop for both regular and irregular forms.

If holistic word representations were to emerge with further training we might expect trained items to activate anterior fusiform regions more than untrained items, in a similar manner to real as well as trained objects relative to words in the current study. In contrast, untrained words might activate left posterior occipitotemporal, parietal, and frontal regions more than trained words, in a similar way to pseudowords relative to real words. This would support neuroimaging studies that, in line with cognitive models of reading, have suggested a distinction between sub- and whole-word processes in dorsal versus ventral brain regions (Taylor et al., 2013). Note however, that although the DRC model proposes lexical orthography-to-phonology mappings, the triangle model (Plaut et al., 1996) proposes that such item-specific mappings primarily emerge for the mapping between visual/phonological word forms and their meanings. Thus, this model might predict activation for trained relative to untrained items in anterior fusiform only if artificial words were trained with meanings.

### 4.3 Parietal and frontal contributions to reading and naming

In addition to vOT contributions, there is substantial evidence for frontal and parietal regions supporting componential reading processes. For example, we saw extensive activation of parietal and frontal networks for the contrast of pseudoword>word reading in the localiser scan. This replicates a large number of previous observations in the functional imaging literature (see Taylor et al., 2013 for a meta-analysis). We will discuss the implications of these findings separately, first for parietal and then for frontal regions.

The observed activation increase in inferior parietal cortices for word reading over object naming replicates results previously reported by Taylor et al. (2014a). However, our results go beyond these in two ways. First, by demonstrating substantial overlap between activation contrasts for artificial and real language stimuli (as scanned during a functional localiser). Second, by showing that differences in parietal involvement can be seen in an event-related design in which trials presenting artificial words and objects are randomly intermixed, rather than the blocked presentation used by Taylor et al. (2014a). This suggests that activation differences can be evoked solely by stimulus differences in the absence of the more strategic effects that are possible for blocked designs.

Unlike Taylor et al. (2014a), we did not find an interaction between item type (words vs objects) and trial type (see-think vs see-speak) in parietal regions. We cannot therefore be certain that parietal involvement in reading words reflects the retrieval of written word pronunciations, independent of the perceptual differences between words and objects. Indeed, some have primarily associated parietal activity during reading with perceptual processes. For example Cohen et al. (2008) using fMRI, and Rosazza et al. (2009) using EEG, argued that parietal regions are primarily active when readers are forced into an “attention-based serial reading strategy” (p 361 of Cohen et al.) by changes in the visual form (orientation or degradation) of written words. A similar, serial visual processing, interpretation was offered by Protopapas et al. (2016) who obtained length effects in this region during pseudoword reading.

However, other data argue against a purely visuo-spatial attention account of parietal activation during reading. Carreiras et al. (2014) demonstrated selectivity in left inferior and superior parietal regions for coding the identity and positions of letters, relative to symbols and numbers, suggesting a role for this region in processing stimuli that have linguistic associations. Parietal activation has also been shown to be greater when participants make judgements about spelling-to-sound mappings, over and above judgements about spellings or sounds in isolation, again implicating this region in cross-modal processing (Booth et al., 2003, Booth et al., 2007). Thus, in line with the proposal made by Taylor et al., 2013, Taylor et al., 2014a, we suggest that engagement of parietal regions for pseudoword relative to word reading, and for artificial word reading relative to object naming, reflects their role in the translation of component letters into sounds.

In our study, posterior frontal (precentral gyrus) and parietal regions largely showed the same response profile. Both regions were activated in the localiser for pseudowords vs real words, and in the contrast of artificial words vs objects. Previous studies have implicated these frontal regions (specifically the precentral gyrus) in the selection and assembly of phonological outputs (Bookheimer, 2002, Devlin et al., 2003, Gough et al., 2005). Left precentral gyrus not only shows increased activation for pseudoword compared to word reading (Taylor et al., 2013, Taylor et al., 2014b), but also reduced activation following consolidation of new spoken words (Davis et al., 2009). Furthermore, a recent fMRI study has dissociated posterior frontal regions (such as the precentral gyrus) that contribute to phonological output from more anterior inferior frontal regions, which may contribute to phonological selection (this latter process is particularly engaged by reading irregular words, Taylor et al., 2014b). This distinction between phonological selection and phonological assembly is consistent with our finding of increased activation for naming artificial objects than reading artificial words in left IFG orbitalis, and the reverse profile in left precentral gyrus.

### 4.4 Emergence of holistic representations for consolidated items

As mentioned above, even after a night of consolidation for day 1 words we did not find differences between trained and untrained words either in behavioural performance or in the regions of interest defined from the functional localiser. Nonetheless, there was some evidence of whole-word learning since the hippocampus was differentially active for items learned on days 1 and 2. Consistent with the predictions of CLS accounts, there was more hippocampal activation for both words and objects learned on the same day as scanning as compared to the previous day. As the words from days 1 and 2 share the same letters, this reduced activation for day 1 words would not be possible unless a whole-word representation of some form existed. Hence, evidence that consolidation impacts on hippocampal activation provides evidence for some form of holistic representation for the day 1 words. However, given the lack of reliable activation differences between trained and untrained written words we cannot be confident about where these representations reside. Further studies should extend and adapt the time period of training and scanning to answer this question. Further work could then also determine the relative roles of sleep and time in the consolidation of orthographic and lexical representations. This question concerning the role of item-level consolidation in early stages of learning to read has implications for the role of consolidation in literacy learning more generally. Although lexical consolidation has been shown for school-age children (Henderson et al., 2013), this has primarily been in the context of learning spoken and not written words.

### 4.5 Validation of laboratory-based learning paradigms and implications for education

The extensive overlap of activation between the functional localiser and the artificial items, combined with the behavioural evidence, shows that laboratory studies of reading can engage holistic and componential learning mechanisms. This is a striking finding when we consider that we are comparing words and objects that have been used since childhood with artificial items that have been learned at most one day prior to scanning. This outcome suggests that laboratory-based learning studies can activate the same neural systems as engaged in more ecologically-valid paradigms (e.g. in studies of beginning readers). However, our artificial orthography studies have an advantage of maintaining strict experimental control. Neuroimaging results for real language stimuli may be sensitive to a wide variety of linguistic features: word frequency, age-of-acquisition, etc. which can be readily controlled in laboratory learning studies. Similarly, real language stimuli may be subject to individual differences in terms of language and literacy exposure that can be readily controlled in a laboratory setting. Finally, educational questions about early literacy acquisition are dependent on a wide range of external factors that may influence classroom outcomes. Consequently we suggest laboratory-based approaches offer an important complement to more naturalistic studies.

In education, the relative importance of holistic and componential reading strategies is much debated, with componential phonics-based approaches to reading instruction being dominant. Our results are consistent with the importance of componential learning during the earliest stages of reading acquisition: we see activation in posterior vOT, parietal, and frontal regions for the contrast of artificial word greater than object naming. However, in contrast to imaging findings with skilled readers and English words we see relatively little activation evidence for holistic representations of artificial written words despite abundant evidence for holistic representation of novel objects.

While fronto-parietal activation has been implicated in many studies of word reading, the relative contributions of dorsal and ventral regions across different stages of learning to read has remained relatively unclear, with several studies citing the need to further investigate the relationship between parietal and vOT contributions (Carreiras et al., 2014, Cohen et al., 2008, Price, 2012; Reilhac et al., 2013). Although fMRI studies of children have shown parietal involvement (Bitan et al., 2007a, Cao et al., 2006, Hoeft et al., 2007), children old enough to undergo scanning already have several years of exposure to written words. By tracking the very earliest stages of learning to read, our laboratory-based approach offers a way to identify the contributions of parietal and vOT regions over the very earliest stages of learning to read. That these areas show extensive activation prior to neural evidence for holistic representations speaks to the important role of componential mechanisms in early stages of acquisition. Given functional imaging and neuropsychological evidence for parietal contributions to spatial encoding of written strings (Carreiras et al., 2014, Cohen et al., 2008), our findings motivate further work to explore parietal contributions to successful and unsuccessful literacy acquisition (Peyrin et al., 2011, Reilhac et al., 2013).

## Figures and Tables

**Table:** 

A1	A2	B1	B2	C1	C2
mAf	dAs	bAv	fAp	vAd	vAk
bAb	fAk	pAz	vAg	dAt	pAf
pAg	zAm	nAm	fAm	gAk	zAt
bAz	pAb	zAd	sAb	tAm	kAs
fAv	zEt	tAf	bEz	nEg	pAv
dEm	gEn	sEg	kEt	bEm	kEb
kEs	dEz	bEp	nEf	tEz	sEp
zEk	vEg	vEd	dEv	fEg	bEv
tEp	gEb	sEk	tEk	mEk	gEd
fQm	mQt	gQp	dQv	pQn	gQm
nQs	sQf	zQn	pQs	kQv	fQd
vQg	bQv	fQt	tQn	zQp	dQz
sQn	pQf	mQn	zQs	nQf	vQn
tQz	kVv	pVm	mQd	dQb	tVs
sVt	tVd	dVs	gVk	mVs	nVg
nVd	vVp	vVt	mVb	fVb	mVn
mVp	nVk	gVb	kVz	sVf	zVt
gVd	kVn	kVg	nVf	bVp	sVz

**Table:** 

Real words			Pseudowords		
ant	dress	nut	abe	dod	nolt
arm	duck	owl	arn	dutt	orn
axe	ear	peach	ath	eash	paunt
ball	egg	pear	bame	eck	pim
bath	eye	pen	barse	elt	plean
bear	fence	pig	bave	farb	pouse
bed	fish	pipe	beb	feach	proo
bee	flag	plug	berve	fedge	pudge
bell	flute	pot	bim	fet	purf
belt	fly	ring	bive	fick	rass
bird	foot	rope	bleer	fiss	remp
book	fork	screw	bolve	flum	sadge
boot	fox	seal	bouth	fuke	scark
bow	frog	sheep	breb	fune	shig
bowl	glass	shirt	brime	gamb	sile
box	glove	shoe	brip	garge	skine
bread	goat	skirt	browl	geg	snay
broom	grapes	skunk	bue	gope	snud
bus	gun	sled	burl	gow	sog
cake	hair	snail	cang	hame	solk
cap	hand	snake	chag	heek	souch
car	harp	sock	ched	helt	spab
cat	hat	spoon	chemp	hong	spale
chain	heart	star	chone	hoose	spape
chair	hook	stool	clag	hosh	spen
church	horse	stove	clope	hount	stonk
clock	house	sun	cly	hudge	strang
cloud	key	swan	cose	kib	strine
clown	kite	swing	crad	kinge	swart
coat	knife	tail	crame	kouse	tark
comb	lamp	thumb	crench	lerge	teb
couch	leaf	tie	crub	lomp	thep
cow	leg	toe	culk	loof	tilm
crown	lips	top	cupe	losh	torb
cup	lock	train	cusk	lun	touth
deer	map	tree	dack	modge	turb
desk	moon	tree	dag	moise	tute
dog	mouse	vase	dalve	mooth	vone
doll	nail	watch	dask	netch	womp
door	nose	wheel	detch	nink	wug

**Table 1:** Behavioural performance.

Performance during training: mean proportion correct (SD) averaged over blocks						
	Artificial objects	Artificial words				
Day 1 training	0.77 (0.13)	0.67 (0.13)				
Day 2 training	0.79 (0.19)	0.86 (0.12)				

Performance during scanning: mean proportion correct (SD)						
	Artificial objects	Artificial words		Written objects	Untrained words	
Trained on day 1	0.51 (0.50)	0.82 (0.38)		0.82 (0.38)		
Trained on day 2	0.63 (0.48)	0.82 (0.38)		0.82 (0.38)		
Untrained					0.80 (0.40)	

Performance during localiser						
	Mean accuracy (*SD*)		Mean RT (*SD*)			
English words	99.04 (2.67)		616.78 (112.81)			
Real objects	99.72 (3.43)		904.58 (238.45)			
Pseudowords	99.12 (2.19)		679.48 (158.34)			

**Table 2:** Brain regions involved in reading real words, objects, and pseudowords in the localiser, thresholded at voxelwise p<0.001 uncorrected, cluster extent corrected. The table reports the first three peaks more than 8 mm apart in each cluster.

Brain region (AAL)	Hemisphere	x	y	z	Voxels	Z Value	Cluster-level p Value
**Words – Objects** (corrected for RT differences)							
Inferior parietal cortex	R	56	−50	42	9314	5.45	<0.001
Supramarginal gyrus		64	−42	30			
Supramarginal gyrus		56	−38	44			
Cuneus	R	14	−70	36	7415	5.39	<0.001
Precuneus		8	−52	36			
White matter		2	−38	24			
Cerebellum	L	−36	−66	−50	1013	5.19	<0.001
Cerebellum		−8	−84	−40			
Cerebellum		−36	−78	−40			
Superior temporal gyrus	L	−48	−32	8	4985	5.16	<0.001
Superior temporal gyrus		−58	−6	6			
White matter		−40	−30	8			
Inferior frontal opercularis	R	54	12	10	401	4.42	0.001
Insula		42	12	−12			
Insula		50	12	−4			
Middle frontal gyrus	L	−26	34	26	393	4.31	0.001
Middle frontal gyrus		−32	36	36			
Middle frontal gyrus		−28	28	32			
Brain region (AAL)	Hemisphere	x	y	z	Voxels	Z Value	Cluster-level p Value

**Objects – Words** (corrected for RT differences)							
Inferior temporal gyrus	R	54	−62	−12	5021	6.02	<0.001
Middle occipital gyrus		36	−88	10			
Inferior occipital gyrus		46	−82	0			
Middle occipital gyrus	L	−24	−94	8	3395	5.27	<0.001
Inferior occipital gyrus		−48	−62	−14			
Middle occipital gyrus		−36	−84	12			
Brain region (AAL)	Hemisphere	x	y	z	Voxels	Z Value	Cluster-level p Value

**Words – Pseudowords**							
Middle temporal gyrus	L	−56	−54	20	1893	5.5	<0.001
Middle occipital gyrus		−40	−78	22			
Middle occipital gyrus		−38	−78	36			
Middle temporal gyrus	R	56	−58	14	1887	5.41	<0.001
Middle temporal gyrus		42	−62	16			
Middle occipital gyrus		44	−62	28			
Cerebellum	L	−8	−56	−44	4312	5.4	<0.001
Cerebellum		−14	−46	−42			
Middle cingulum		−8	−34	40			
Fusiform gyrus	L	−36	−36	−10	315	4.8	0.002
Fusiform gyrus		−28	−38	−16			
Fusiform gyrus		−26	−30	−24			
Middle frontal gyrus	L	−28	30	46	470	4.47	<0.001
Middle frontal gyrus		−32	28	38			
Superior frontal gyrus		−24	38	44			
Superior frontal gyrus	R	24	34	46	663	4.45	<0.001
Superior frontal gyrus		28	24	52			
Middle frontal gyrus		24	24	42			
Cerebellum	R	14	−46	−42	160	4.33	0.049
Cerebellum		−12	−52	−46			
Cerebellum		−4	−54	−44			
Medial frontal orbitalis	R	2	54	0	202	3.75	0.02
Medial frontal orbitalis		4	46	0			
Brain region (AAL)	Hemisphere	x	y	z	Voxels	Z Value	Cluster-level p Value

**Pseudoword – Words**							
Cerebellum	R	36	−64	−22	1362	5.26	<0.001
Middle occipital gyrus		44	−82	2			
Inferior occipital gyrus		36	−88	−2			
Superior temporal gyrus	L	−62	−18	6	3930	4.96	<0.001
Precentral gyrus		−56	4	26			
Postcentral gyrus		−60	−18	20			
Inferior temporal gyrus	L	−42	−60	−8	802	4.6	<0.001
Middle occipital gyrus		−30	−94	−4			
Inferior occipital gyrus		−40	−74	−8			
Supplementary motor area	L	−4	2	60	205	4.3	0.019
Postcentral gyrus	R	66	−10	34	259	4.02	0.006
Precentral gyrus		62	4	28			
Precentral gyrus		46	−10	40			
Inferior frontal triangularis	L	−48	36	16	192	3.8	0.025
Inferior frontal triangularis		−40	36	14			
Inferior frontal triangularis		−42	34	6			

**Table 3:** Brain regions involved in reading artificial words and naming artificial objects.

Brain region (AAL)	Hemisphere	x	y	z	Voxels	Z Value	Cluster-level p Value
Words – Objects, collapsed across Day and See-Think/See-Speak condition, correct responses only							
Superior parietal cortex	R	24	−62	54	6171	Inf	<0.001
Supramarginal gyrus		46	−30	44			
Inferior temporal gyrus		50	−60	−8			
Superior parietal cortex	L	−24	−60	52	14011	Inf	<0.001
Inferior parietal cortex		−40	−38	40			
Middle occipital gyrus		−28	−68	26			
Cerebellum	R	26	−70	−50	1817	7.13	<0.001
Cerebellum		26	−64	−26			
Cerebellum		6	−70	−24			
White matter	L & R	0	22	4	668	5.72	<0.001
White matter		16	30	6			
White matter		−16	30	2			
Precentral gyrus	R	48	6	30	416	5.53	<0.001
Brain region (AAL)	Hemisphere	x	y	z	Voxels	Z Value	Cluster-level p Value

Objects – Words, collapsed across Day and See-Think/See-Speak condition, correct responses only							
Angular gyrus	L	−48	−64	40	3799	Inf	<0.001
Middle occipital gyrus		−40	−72	36			
Middle temporal gyrus		−58	−24	−12			
Angular gyrus	R	44	−62	38	1969	7.57	<0.001
Angular gyrus		54	−58	26			
Inferior parietal cortex		54	−56	46			
Middle cingulum	L	−2	−40	36	3890	7.34	<0.001
Precuneus		−8	−66	32			
Precuneus		−6	−60	16			
Middle frontal gyrus	L	−38	16	48	10405	6.98	<0.001
Medial superior frontal gyrus		−4	42	30			
Medial superior frontal gyrus		−36	56	0			
Fusiform gyrus	L	−28	−46	−14	1177	6.38	<0.001
Lingual		−28	−84	−14			
Lingual		−24	−96	−12			
Fusiform gyrus	R	30	−54	−10	1046	5.98	<0.001
Fusiform gyrus		32	−74	−12			
Fusiform gyrus		28	−42	−14			
Cerebellum	R	18	−84	−34	446	5.45	0.001
Cerebellum		44	−60	−42			
Cerebellum		46	−68	−40			
Inferior temporal gyrus	R	58	−24	−16	687	5.26	<0.001
Middle temporal gyrus		48	−36	−2			
Calcarine fissure	R	22	−100	−4	301	5.24	0.007
Cuneus		12	−98	16			
Calcarine fissure		14	−102	4			
Brain region (AAL)	Hemisphere	x	y	z	Voxels	Z Value	Cluster-level p Value

Words [see-think - see-speak] − Objects [see-think − see-speak], collapsed across Day, correct responses only							
Medial superior frontal gyrus	R	12	58	30	648	4.23	<0.001
Superior frontal gyrus		−14	58	30			
Medial superior frontal gyrus		10	64	14			
Brain region (AAL)	Hemisphere	x	y	z	Voxels	Z Value	Cluster-level p Value

Objects [see-think − see-speak] - Words [see-think − see-speak], collapsed across Day, correct responses only							
Fusiform gyrus	R	32	−52	−16	1478	6.6	<0.001
Fusiform gyrus		32	−70	−12			
Fusiform gyrus		36	−32	−22			
Fusiform gyrus	L	−30	−46	−20	1021	6.83	<0.001
Cuneus	L	−12	−64	30	324	5.05	0.005
Inferior parietal cortex	L	−44	−54	46	223	4.59	0.026

*p*<0.001 and cluster-level FWE-corrected at p<0.05. All peaks >8 mm apart are reported.

**Table 4:** Region of interest analyses.

Region	Centre of Mass			Item type (word vs objects)			Trial type (see-think vs see-speak)			Interaction	
	X	Y	Z	F(1,19)	ŋ_p_^2^		F(1,19)	ŋ_p_^2^		F(1,19)	ŋ_p_^2^
Anterior fusiform	−28	−50	−16	33.92***	0.64	Objects>Words	0.45			22.15***	0.54
Left posterior vOT	−42	−60	−8	15.60***	0.45	Words>Objects	44.34***	0.70	See-only>see-speak	1.14	
Precentral gyrus	−56	4	26	28.43***	0.60	Words>Objects	12.94**	0.40	See-speak>see-only	0.01	
Superior parietal lobe	−26	−60	54	53.79***	0.74	Words>Objects	6.73*	0.26	See-only>see-speak	0.14	
				
Region	Centre of Mass			Reading generalisation conditions							
	X	Y	Z	F(4,76)							
Anterior fusiform	−28	−50	−16	0.709							
Left posterior vOT	−42	−60	−8	0.364							
Precentral gyrus	−56	4	26	0.319							
Superior parietal lobe	−26	−60	54	0.063							

* p<0.05.

** p<0.01.

*** p<0.001.

## References

[bib1] Anderson M.C. (2003). Rethinking interference theory: executive control and the mechanisms of forgetting. J. Mem. Lang..

[bib2] Ashburner J., Friston K.J. (2005). Unified segmentation. Neuroimage.

[bib3] Ben-Shachar M., Dougherty R.F., Deutsch G.K., Wandell B.A. (2011). The development of cortical sensitivity to visual word forms. J. Cogn. Neurosci..

[bib4] Bitan T., Burman D.D., Lu D., Cone N.E., Gitelman D.R., Mesulam M.-M., Booth J.R. (2006). Weaker top–down modulation from the left inferior frontal gyrus in children. Neuroimage.

[bib5] Bitan T., Burman D.D., Chou T.-L., Lu D., Cone N.E., Cao F., Booth J.R. (2007). The interaction between orthographic and phonological information in children: an fMRI study. Hum. Brain Mapp..

[bib6] Bitan T., Cheon J., Lu D., Burman D.D., Gitelman D.R., Mesulam M.-M., Booth J.R. (2007). Developmental changes in activation and effective connectivity in phonological processing. Neuroimage.

[bib7] Bookheimer S. (2002). Functional MRI of language: new approaches to understanding the cortical organization of semantic processing. Annu. Rev. Neurosci..

[bib8] Booth J.R., Cho S., Burman D.D., Bitan T. (2007). Neural correlates of mapping from phonology to orthography in children performing an auditory spelling task. Dev. Sci..

[bib9] Booth J.R., Burman D.D., Meyer J.R., Gitelman D.R., Parrish T.B., Mesulam M. (2003). Relation between brain activation and lexical performance. Hum. Brain Mapp..

[bib10] Breitenstein C., Jansen A., Deppe M., Foerster A.-F., Sommer J., Wolbers T., Knecht S. (2005). Hippocampus activity differentiates good from poor learners of a novel lexicon. Neuroimage.

[bib11] Brem S., Bach S., Kucian K., Kujala J.V., Guttorm T.K., Martin E., Richardson U. (2010). Brain sensitivity to print emerges when children learn letter–speech sound correspondences. Proc. Natl. Acad. Sci..

[bib12] Cao F., Brennan C., Booth J.R. (2015). The brain adapts to orthography with experience: evidence from English and Chinese. Dev. Sci..

[bib13] Cao F., Bitan T., Chou T.-L., Burman D.D., Booth J.R. (2006). Deficient orthographic and phonological representations in children with dyslexia revealed by brain activation patterns. J. Child Psychol. Psychiatry.

[bib14] Carreiras M., Quiñones I., Hernández-Cabrera J.A., Duñabeitia J.A. (2014). Orthographic coding: brain activation for letters, symbols, and digits. Cereb. Cortex..

[bib15] Carreiras M., Seghier M.L., Baquero S., Estévez A., Lozano A., Devlin J.T., Price C.J. (2009). An anatomical signature for literacy. Nature.

[bib16] Cattinelli I., Borghese N.A., Gallucci M., Paulesu E. (2013). Reading the reading brain: a new meta-analysis of functional imaging data on reading. J. Neurolinguist..

[bib17] Church J.A., Coalson R.S., Lugar H.M., Petersen S.E., Schlaggar B.L. (2008). A developmental fMRI study of reading and repetition reveals changes in phonological and visual mechanisms over age. Cereb. Cortex.

[bib18] Cohen L., Dehaene S., Vinckier F., Jobert A., Montavont A. (2008). Reading normal and degraded words: contribution of the dorsal and ventral visual pathways. Neuroimage.

[bib19] Cohen L., Lehéricy S., Chochon F., Lemer C., Rivaud S., Dehaene S. (2002). Language-specific tuning of visual cortex? Functional properties of the visual word form area. Brain.

[bib20] Cohen L., Dehaene S., Naccache L., Lehéricy S., Dehaene-Lambertz G., Hénaff M.-A., Michel F. (2000). The visual word form area. Brain.

[bib21] Coltheart M., Rastle K., Perry C., Langdon R., Ziegler J. (2001). DRC: a dual route cascaded model of visual word recognition and reading aloud. Psychol. Rev..

[bib22] Cusack R., Vicente-Grabovetsky A., Mitchell D.J., Wild C.J., Auer T., Linke A.C., Peelle J.E. (2014). Automatic analysis (aa): efficient neuroimaging workflows and parallel processing using Matlab and XML. Front. Neuroinformatics.

[bib23] Davis M.H., Gaskell M.G. (2009). A complementary systems account of word learning: neural and behavioural evidence. Philos. Trans. R. Soc. B: Biol. Sci..

[bib24] Davis M.H., Di Betta A.M., Macdonald M.J., Gaskell M.G. (2009). Learning and consolidation of novel spoken words. J. Cogn. Neurosci..

[bib25] Dehaene S., Cohen L. (2007). Cultural recycling of cortical maps. Neuron.

[bib26] Dehaene S., Cohen L. (2011). The unique role of the visual word form area in reading. Trends Cogn. Sci..

[bib27] Dehaene S., Cohen L., Sigman M., Vinckier F. (2005). The neural code for written words: a proposal. Trends Cogn. Sci..

[bib28] Dehaene S., Cohen L., Morais J., Kolinsky R. (2015). Illiterate to literate: behavioural and cerebral changes induced by reading acquisition. Nat. Rev. Neurosci..

[bib29] Dehaene S., Le Clec’H G., Poline J.-B., Le Bihan D., Cohen L. (2002). The visual word form area: a prelexical representation of visual words in the fusiform gyrus. Neuroreport.

[bib30] Dehaene S., Pegado F., Braga L.W., Ventura P., Nunes F., Jobert A., Cohen L. (2010). How learning to read changes the cortical networks for vision and language. Science.

[bib31] Devlin J.T., Matthews P.M., Rushworth M.F. (2003). Semantic processing in the left inferior prefrontal cortex: a combined functional magnetic resonance imaging and transcranial magnetic stimulation study. J. Cogn. Neurosci..

[bib32] Friston K.J., Ashburner J., Frith C.D., Poline J.-B., Heather J.D., Frackowiak R.S. (1995). Spatial registration and normalization of images. Hum. Brain Mapp..

[bib33] Glezer L.S., Jiang X., Riesenhuber M. (2009). Evidence for highly selective neuronal tuning to whole words in the “visual word form area. Neuron.

[bib34] Gough P.M., Nobre A.C., Devlin J.T. (2005). Dissociating linguistic processes in the left inferior frontal cortex with transcranial magnetic stimulation. J. Neurosci..

[bib35] Gusnard D.A., Raichle M.E. (2001). Searching for a baseline: functional imaging and the resting human brain. Nat. Rev. Neurosci..

[bib36] Hagoort P., Indefrey P., Brown C., Herzog H., Steinmetz H., Seitz R.J. (1999). The neural circuitry involved in the reading of German words and pseudowords: a PET study. J. Cogn. Neurosci..

[bib37] Henderson L., Weighall A., Gaskell G. (2013). Learning new vocabulary during childhood: effects of semantic training on lexical consolidation and integration. J. Exp. Child Psychol..

[bib38] Henson R.N., Penny W. (2003). ANOVAs and SPM. Technical report. Wellcome Dep. Imaging Neurosci..

[bib39] Hoeft F., Meyler A., Hernandez A., Juel C., Taylor-Hill H., Martindale J.L., Gabrieli J.D. (2007). Functional and morphometric brain dissociation between dyslexia and reading ability. Proc. Natl. Acad. Sci..

[bib40] James T.W., Humphrey G.K., Gati J.S., Menon R.S., Goodale M.A. (2002). Differential effects of viewpoint on object-driven activation in dorsal and ventral streams. Neuron.

[bib41] Josephs O., Henson R.N. (1999). Event-related functional magnetic resonance imaging: modelling, inference and optimization. Philos. Trans. R. Soc. B: Biol. Sci..

[bib42] Kherif F., Josse G., Price C.J. (2011). Automatic top-down processing explains common left occipito-temporal responses to visual words and objects. Cereb. Cortex.

[bib43] Loftus G.R., Masson M.E. (1994). Using confidence intervals in within-subject designs. Psychon. B. Rev..

[bib44] MacLeod C.M. (1991). Half a century of research on the Stroop effect: an integrative review. Psychol. Bull..

[bib45] Magnié M.N., Besson M., Poncet M., Dolisi C. (2003). The Snodgrass and Vanderwart set revisited: norms for object manipulability and for pictorial ambiguity of objects, chimeric objects, and nonobjects. J. Clin. Exp. Neuropsychol..

[bib46] Martin A., Schurz M., Kronbichler M., Richlan F. (2015). Reading in the brain of children and adults: a meta-analysis of 40 functional magnetic resonance imaging studies. Hum. Brain Mapp..

[bib47] Mechelli A., Crinion J.T., Long S., Friston K.J., Ralph M., Patterson K., Price C. (2005). Dissociating reading processes on the basis of neuronal interactions. J. Cogn. Neurosci..

[bib48] Mei L., Xue G., Lu Z.-L., He Q., Zhang M., Xue F., Dong Q. (2013). Orthographic transparency modulates the functional asymmetry in the fusiform cortex: an artificial language training study. Brain Lang..

[bib49] Mensink G.-J., Raaijmakers J.G. (1988). A model for interference and forgetting. Psychol. Rev..

[bib50] Peelle J.E. (2014). Methodological challenges and solutions in auditory functional magnetic resonance imaging. Front. Neurosci..

[bib51] Perrachione T.K., Ghosh S.S. (2013). Optimized design and analysis of sparse-sampling fMRI experiments. Front. Neurosci..

[bib52] Peyrin C., Démonet J.F., N’Guyen-Morel M.A., Le Bas J.F., Valdois S. (2011). Superior parietal lobule dysfunction in a homogeneous group of dyslexic children with a visual attention span disorder. Brain Lang..

[bib53] Plaut D.C., McClelland J.L., Seidenberg M.S., Patterson K. (1996). Understanding normal and impaired word reading: computational principles in quasi-regular domains. Psychol. Rev..

[bib54] Price C.J. (2012). A review and synthesis of the first 20 years of PET and fMRI studies of heard speech, spoken language and reading. Neuroimage.

[bib55] Price C.J., Devlin J.T. (2011). The interactive account of ventral occipitotemporal contributions to reading. Trends Cogn. Sci..

[bib56] Price C.J., Wise R.J.S., Frackowiak R.S.J. (1996). Demonstrating the Implicit Processing of Visually Presented Words and Pseudowords. Cereb. Cortex.

[bib57] Protopapas A., Orfanidou E., Taylor J.S.H., Karavasilis E., Kapnoula E.C., Panagiotaropoulou G., Kelekis D. (2016). Evaluating cognitive models of visual word recognition using fMRI: effects of lexical and sublexical variables. NeuroImage.

[bib58] Raichle M.E. (2015). The brain's default mode network. Annu. Rev. Neurosci..

[bib59] Rastle K., Coltheart M. (1999). Serial and strategic effects in reading aloud. J. Exp. Psychol.: Hum. Percept. Perform..

[bib60] Rastle K., Harrington J., Coltheart M. (2002). 358,534 nonwords: the ARC nonword database. Q. J. Exp. Psychol.: Sect. A.

[bib61] Rastle K., Croot K.P., Harrington J.M., Coltheart M. (2005). Characterizing the motor execution stage of speech production: consonantal effects on delayed naming latency and onset duration. J. Exp. Psychol.: Hum. Percept. Perform..

[bib62] Reilhac C., Peyrin C., Démonet J.-F., Valdois S. (2013). Role of the superior parietal lobules in letter-identity processing within strings: fmri evidence from skilled and dyslexic readers. Neuropsychologia.

[bib63] Rosazza C., Cai Q., Minati L., Paulignan Y., Nazir T.A. (2009). Early involvement of dorsal and ventral pathways in visual word recognition: an ERP study. Brain Res..

[bib64] Rose, J., 2006. Independent review of the teaching of early Reading, Final Report, London, England: Department for Education and Skills.

[bib65] Seghier M.L., Lee H.L., Schofield T., Ellis C.L., Price C.J. (2008). Inter-subject variability in the use of two different neuronal networks for reading aloud familiar words. Neuroimage.

[bib66] Song Y., Bu Y., Hu S., Luo Y., Liu J. (2010). Short-term language experience shapes the plasticity of the visual word form area. Brain Res..

[bib67] Takashima A., Wagensveld B., Van Turennout M., Zwitserlood P., Hagoort P., Verhoeven L. (2014). Training-induced neural plasticity in visual-word decoding and the role of syllables. Neuropsychologia.

[bib68] Taylor J.S.H., Rastle K., Davis M.H. (2013). Can cognitive models explain brain activation during word and pseudoword reading? A meta-analysis of 36 neuroimaging studies. Psychol. Bull..

[bib69] Taylor J.S.H., Rastle K., Davis M.H. (2014). Distinct neural specializations for learning to read words and name objects. J. Cogn. Neurosci..

[bib70] Taylor J.S.H., Rastle K., Davis M.H. (2014). Interpreting response time effects in functional imaging studies. NeuroImage.

[bib71] Torgerson C., Brooks G., Hall J. (2006). A systematic review of the research literature on the use of phonics in the teaching of reading and spelling.

[bib72] Turkeltaub P.E., Flowers D.L., Lyon L.G., Eden G.F. (2008). Development of ventral stream representations for single letters. Ann. N.Y. Acad. Sci..

[bib73] Twomey T., Duncan K.J.K., Price C.J., Devlin J.T. (2011). Top-down modulation of ventral occipito-temporal responses during visual word recognition. Neuroimage.

[bib74] Van Heuven W.J.B., Mandera P., Keuleers E., Brysbaert M. (2014). Subtlex-UK: a new and improved word frequency database for British English. Q. J. Exp. Psychol..

[bib75] Vinckier F., Dehaene S., Jobert A., Dubus J.P., Sigman M., Cohen L. (2007). Hierarchical coding of letter strings in the ventral stream: dissecting the inner organization of the visual word-form system. Neuron.

[bib76] Vuilleumier P., Henson R.N., Driver J., Dolan R.J. (2002). Multiple levels of visual object constancy revealed by event-related fMRI of repetition priming. Nat. Neurosci..

[bib77] Wyse D., Goswami U. (2008). Synthetic phonics and the teaching of reading. Br. Educ. Res. J..

[bib78] Yoncheva Y.N., Wise J., McCandliss B. (2015). Hemispheric specialization for visual words is shaped by attention to sublexical units during initial learning. Brain Lang..

[bib79] Yoncheva Y.N., Blau V.C., Maurer U., McCandliss B.D. (2010). Attentional focus during learning impacts N170 ERP responses to an artificial script. Dev. Neuropsychol..

